# The Dose-Response Relationship between Gamma-Glutamyl Transferase and Risk of Diabetes Mellitus Using Publicly Available Data: A Longitudinal Study in Japan

**DOI:** 10.1155/2020/5356498

**Published:** 2020-02-21

**Authors:** Wei Zhao, Jingjing Tong, Jie Liu, Jin Liu, Jinghua Li, Yongtong Cao

**Affiliations:** ^1^Department of Clinical Laboratory, China-Japan Friendship Hospital, Beijing 100029, China; ^2^Liver Failure Treatment and Research Center, The Fifth Medical Center of PLA General Hospital, Beijing 100039, China; ^3^Department of Vascular and Endovascular Surgery, Chinese PLA General Hospital, Beijing 100853, China; ^4^Watson Longcheng Technology and Trade Co., Ltd., Beijing 100020, China

## Abstract

**Purpose:**

The purpose of this study was to examine the association between baseline serum gamma-glutamyl transferase (GGT) and incident diabetes mellitus and to explore their dose-response relationship in a cohort of Japanese adults. *Patients and Methods*. Data were drawn from the NAGALA (NAfld in the Gifu Area, Longitudinal Analysis) study between 2004 and 2015, including hierarchical information on participants ≥18 years of age without diabetes mellitus, preexisting diabetes mellitus, heavy alcohol drinking, or other liver diseases (e.g., hepatitis B/C). The final analytic sample included 15464 participants, 373 of who were diagnosed as diabetes mellitus with a maximum 13-year follow-up. The risk of incident diabetes mellitus according to baseline serum GGT was estimated using multivariable Cox proportional hazards models and a two-piecewise linear regression model was developed to find out the threshold effect.

**Results:**

Being in the highest quintile versus the lowest quintile of GGT levels was associated with an almost twofold increased risk of incident diabetes mellitus (hazard ratio 1.83 (95% CI 1.06, 3.15)), independent of age, gender, smoking status, alcohol intake, BMI, SBP, triglycerides, fatty liver, ALT, AST, and fasting plasma glucose. Further analysis revealed a positive curvilinear association between GGT and incident diabetes mellitus, with a saturation effect predicted at 24 IU/L. When serum GGT level was less than 24 IU/L, the risk of developing diabetes mellitus increased significantly with an increase in serum GGT levels (HR 1.04 (1.02, 1.07), *P*=0.0017). Besides, the association was more significant in nonsmoking participants than ex- or current-smokers (*P*=0.0017). Besides, the association was more significant in nonsmoking participants than ex- or current-smokers (*P* for interaction = 0.0378).

**Conclusion:**

Serum GGT level was a significant predictor of subsequent risk of diabetes mellitus, which increased by 4% for every 1 IU/L increase in GGT when GGT was less than 24 IU/L.

## 1. Introduction

According to the International Diabetes Federation, the global number of patients with type 2 diabetes (T2DM) has increased unprecedentedly [1]. There were 425 million people diagnosed with diabetes mellitus around the world in 2015, and this figure is expected to reach 642 million by 2040 [1]. As the most common chronic disease, diabetes mellitus causes huge socioeconomic pressures on both patients and healthcare systems [2]. It is, therefore, important to fully understand risk factors for diabetes mellitus, which can be used to prevent and screen diabetes mellitus.

The liver is the major organ for glucose metabolism and regulation. Gamma-glutamyl transferase (GGT), alanine aminotransferase (ALT), and aspartate aminotransferase (AST) are often used as biomarkers for liver function [3]. GGT is a transferase that catalyzes the transfer of gamma-glutamyl functional groups from glutathione to other acceptors to regulate the redox status and may be a marker of oxidative stress [4], which plays a role in the pathogenesis of T2DM [5].

A number of studies have reported that elevated baseline GGT levels, even within the normal range, are strongly associated with increased risk of T2DM [6–8]. However, the dose-response relationship between baseline GGT levels and risk of diabetes mellitus has not been elaborated clearly. A cross-section study revealed the continuous positive association between GGT and diabetes mellitus without a threshold effect [9]. While in a meta-analysis, it was reported that GGT contributes to an increased risk of T2DM in a nonlinear dose-response pattern [10]. Therefore, the objectives of this cohort study are to examine the association between baseline GGT levels and risk of diabetes mellitus and to characterize the nature of the dose-response relationship in detail.

## 2. Materials and Methods

### 2.1. Data Source

All these date were obtained from the Dryad Digital Repository (https://datadryad.org/). This website permitted users to freely download the raw data. According to Dryad Terms of Service, we cited Dryad data package ([11], “Data from: Ectopic fat obesity presents the greatest risk for incident type 2 diabetes: a population-based longitudinal study,” Dryad, Dataset, https://doi.org/10.5061/dryad.8q0p192) in the present study.

### 2.2. Study Design and Participants

**Figure 1:**
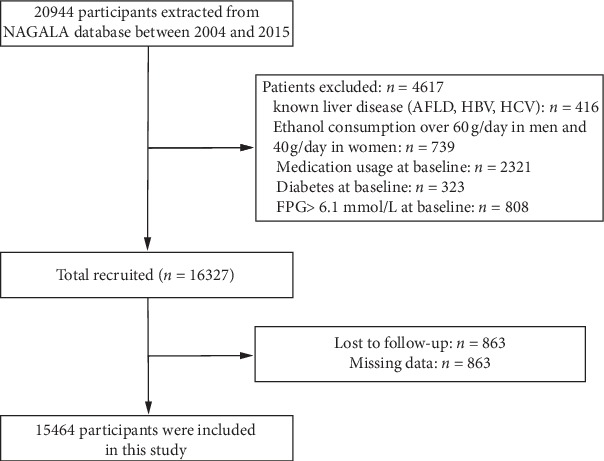
Flow diagram of the screening and enrollment of study participants. Abbreviations: NAGALA, NAfld in the Gifu Area Longitudinal Analysis; AFLD, alcoholic fatty liver disease; HBV, hepatitis B virus; HCV, hepatitis C virus; FPG, fasting plasma glucose.

The NAGALA (NAfld in the Gifu Area, Longitudinal Analysis) study is a population-based longitudinal study at Murakami Memorial Hospital (Gifu, Japan), which was designed to promote public health by detecting chronic diseases and evaluating their risk factors. The details of the NAGALA study were described previously [11, 12]. Briefly, 20944 subjects who participated in medical examination program between 2004 and 2015 and finished a second exam were recruited at Murakami Memorial Hospital. We excluded participants who drank alcohol over 60 g/day for men and 40 g/day for women (*n* = 739), had known liver disease such as hepatitis B or C virus (*n* = 416), had diabetes mellitus (*n* = 323) or impaired fasting glucose (fasting plasma glucose ≥6.1 mmol/L, *n* = 808) at baseline, and used medication at baseline examination (*n* = 2321). Another 863 participants were excluded because of missing data of covariates. The resulting cohort included 15464 participants for the final analysis (Figure 1). Approval was given by the Ethics Committee of Murakami Memorial Hospital, and written informed consent was obtained from all participants of the study.

### 2.3. Data Collection and Measurements

The baseline examination included anthropometric measurements (weight, waist circumference, and blood pressure), blood test, and a questionnaire on the medical history and lifestyle characteristics, including physical activity, smoking, and alcohol habits. Fasting blood samples were analyzed for ALT, AST, GGT, HDL, TC, TG, fasting plasma glucose, and glycated haemoglobin (HbA1c).

As described before [12], alcohol consumption was evaluated by the type and amount of alcoholic beverage consumption per week during the prior month and then calculating the mean alcohol intake per week. Smoking status was divided into three categories: never, past, and current. Body mass index (BMI) was calculated as body weights (kg) divided by square of the participants' heights (m). Abdominal ultrasonography was performed by trained technicians. According to the images, gastroenterologists diagnosed fatty liver without reference to other individual data of the participants. Finally, incident diabetes mellitus was diagnosed according to the American Diabetes Association criteria [13] of self-reported clinician-diagnosed diabetes: fasting plasma glucose ≥7.0 mmol/L or HbA1c ≥ 6.5% or as the initiation of diabetes treatment.

### 2.4. Statistical Analysis

Data are presented as mean ± standard deviation (SD) for continuous variables and as frequency or percentage for categorical variables. For baseline characteristics analysis, the statistical differences among quintiles of GGT were tested with one-way ANOVA for continuous variables and chi-square test for categorical variables. Hazard ratios (HRs) and 95% CIs were calculated for incident diabetes mellitus with serum GGT levels using Cox proportional hazards models. Both nonadjusted and multivariate-adjusted models were used. To assess confounding, we entered covariates into a Cox regression model in the basic model or eliminated the covariates in the complete model one by one and compared the regression coefficients. Those covariates altering initial regression coefficients by more than 10% were included. In this study, the Cox models were adjusted for age, gender, smoking status, alcohol intake, BMI, SBP, triglycerides, fatty liver, ALT, AST, and fasting plasma glucose. Tests for trend were conducted with linear regression by entering the median value of each GGT quintile as a continuous variable in the models.

A generalized additive model was used to assess the nonlinear relationship between serum GGT levels and incident diabetes mellitus. According to the smoothing curve, we further developed a two-piecewise linear regression model to find out the threshold effect, with adjustment for potential confounders. The threshold level of GGT was determined using a recurrence method, including selecting the turning point along a predefined interval and choosing the turning point that yielded the maximum likelihood model. A log-likelihood ratio test was used to compare the two-piecewise linear regression model with the one-line linear model. The subgroup analyses were conducted by alcohol intake, smoking status, BMI, and waist circumference using stratified Cox regression models. Interaction across subgroups was tested using the likelihood ratio test.

For all statistical analyses, we used statistical packages R version 3.4.3 (The R Foundation, Vienna, Austria) and EmpowerStats (X&Y Solutions, Inc., Boston, MA, USA). A two-sided *P* value <0.05 was considered to be statistically significant.

## 3. Results

### 3.1. Baseline Characteristics of Study Participants by Categories of Serum GGT Levels

Among the 15464 participants from the study, 373 subjects were diagnosed as diabetes mellitus with a median 5.4-year follow-up. Baseline characteristics of all participants are shown in Table 1. The mean age was 43.71 ± 8.90 years and almost half of the participants (7034 subjects, 45.49%) were female. The median GGT level was 15 (11–22) IU/L. Participants with higher serum GGT levels were more likely to be older, male, hypertensive, smokers (past and current) and to drink more alcohol. Furthermore, serum GGT level is directly proportional to BMI, waist circumference, fatty liver, ALT, AST, total cholesterol, triglyceride, HbA1c, and fasting plasma glucose levels, while inversely proportional to HDL-cholesterol levels.

### 3.2. Association of Serum GGT Levels with Diabetes Mellitus

Table 2 shows the HRs and 95% CIs for risk of incident diabetes mellitus determined by serum GGT levels. In the non-adjusted model, there was an increasing risk for developing diabetes mellitus as the quintile of GGT increased (*P* for trend <0.0001). Participants who had a concentration of GGT in the highest quintile versus the lowest quintile had a ninefold increased risk in the odds of the development of diabetes mellitus [HR 9.27 (95% CI 5.83, 14.72)]. After adjustment for age, gender, smoking status, alcohol intake, BMI, SBP, fatty liver, ALT, AST, TG and FPG, the hazard ratios were 1.39 (0.77, 2.50), 1.15 (0.66, 1.98), 1.76 (1.04, 3.00), and 1.83 (1.06, 3.15) for GGT quintiles 2–5, respectively (*P* for trend = 0.0097).

### 3.3. Threshold Effect Analysis of GGT on Incident Diabetes Mellitus

**Figure 2:**
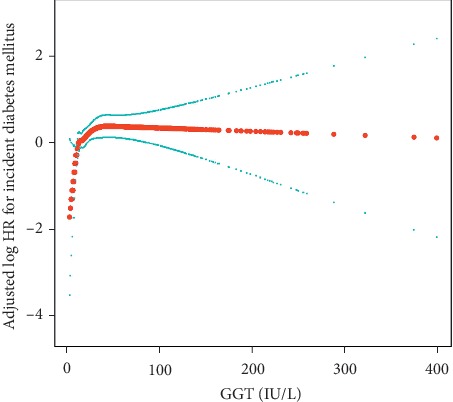
Dose-response relationship between GGT and incident diabetes mellitus in the NAGALA study, 2004–2015.

To evaluate whether a dose-response relationship between GGT and incident diabetes mellitus existed, we used a smoothing function analysis. After adjusting for potential confounding factors, a nonlinear relationship between serum GGT levels and diabetes mellitus was observed (Figure 2). The risk of developing diabetes mellitus was positively correlated with the serum GGT levels until it peaks at 24 IU/L [HR 1.04 (1.02, 1.07), *P*=0.0017]. However, when the concentration of GGT was higher than 24 IU/L, the hazard ratios for risk of developing diabetes mellitus was 1.00 (0.99, 1.01), indicating that the risk of developing diabetes mellitus did not increase significantly with an increase in GGT levels (*P*=0.9288) (Table 3).

In the figure, the red line indicates the estimated risk of incident diabetes mellitus, and the blue lines represent point-wise 95% confidence interval adjusted for age, gender, smoking status, alcohol intake, body mass index, systolic blood pressure, fatty liver, triglycerides, alanine aminotransferase, aspartate aminotransferase, and fasting plasma glucose.

### 3.4. Subgroup Analyses

Smoking, drinking, and obesity are known confounders of GGT-diabetes association. To see if the association between serum GGT levels and incident diabetes mellitus is stable in different subgroups, we did stratified analyses and interactive analyses (Table 4). Data showed that smoking status played an interactive role in the association between GGT and incident diabetes mellitus (*P* for interaction = 0.0378). The associations with being in the top four quintiles of GGT levels were stronger for the participants who never smoked (quintile 2 1.89 (0.90, 3.95); quintile 3 1.40 (0.67, 2.90); quintile 4 2.60 (1.28, 5.27); and quintile 5 2.57 (1.22, 5.39) vs. quartile 1 1.00, *P* for trend = 0.0090). No significant associations were observed among those who smoked in the past or now. Similar results were found in different drinking status, although the differences were not statistically significant (*P* for interaction = 0.2865). It was also observed that the hazard ratios were higher in the participants whose BMI ≥ 25 kg/m^2^, or waist circumference ≥90 in men, ≥80 in women.

## 4. Discussion

In this population-based cohort study, GGT was found to be associated with an elevated risk of the incidence of diabetes mellitus, independent of age, gender, smoking status, alcohol intake, BMI, SBP, ALT, AST, TG, and FPG. We further revealed a nonlinear relationship between serum GGT levels and risk of diabetes mellitus. The relationship was characterized as follows: the risk of developing diabetes mellitus increased significantly with an increase in serum GGT levels when the GGT level was less than 24 IU/L and the risk almost leveled off when the GGT level was beyond 24 IU/L. Interestingly, the significant association was observed in the participants who never smoked, but not in those who smoked in the past or now.

Most of the previous researches examining the associations between concentrations of GGT and incident T2DM reported a positive association [6, 8, 14–19]. Our findings with GGT are consistent with those studies. They also found that GGT, even within normal range, was an important predictor of diabetes mellitus [6, 17, 20]. However, what is the normal range had not been clearly elucidated. Only few studies have examined the dose-response relationship in detail, but produced inconsistent results. In a cross-sectional study of 7976 participants in Singapore [9], it showed that the observed positive association between higher quartiles of GGT and diabetes mellitus was present across the full range of GGT levels, without a threshold effect. Another meta-analysis [10], which included 23 articles based on 24 unique prospective cohort studies, assessed the dose-response relationship between GGT levels and risk of T2DM and found that there was a nonlinear association with a turning point 35 IU/L of serum GGT levels. To our knowledge, this is the first paper systematically studying the dose-response relationship in a cohort study and the results from our investigation confirmed the nonlinear association. The risk of diabetes mellitus increases by 4% for every 1 IU/L increase in GGT when GGT is less than 24 IU/L.

Our subgroup analysis revealed that the association between serum GGT levels and risk of diabetes mellitus stably existed between the layers except smoking and drinking status. It showed that serum GGT concentration was positively associated with incidence of diabetes mellitus in nonsmoking (HR = 2.57 (1.22, 5.39) in GGT quintile 5, *P* for trend = 0.0090) or nondrinking participants (HR = 3.92 (1.60, 9.58) in GGT quintile 5, *P* for trend = 0.0003). However, the associations were not observed in drinkers, ex- or current-smokers. The associations between cigarette smoking and the development of diabetes mellitus have been well demonstrated [21–23]. In the health professionals' follow-up study, men who smoked 25 or more cigarettes daily had a relative risk of diabetes mellitus of 1.94 (1.25, 3.03) compared with nonsmokers [22]. In another prospective study comprising 41372 men and women, it was reported that HRs were 1.22 (1.04, 1.43) among men smoking less than 20 cigarettes daily and 1.57 (1.34, 1.84) among men smoking 20 cigarettes daily or more and in women the corresponding HRs were 1.46 (1.21–1.76) and 1.87 (1.36–2.59), respectively [24]. The probable mechanisms included that (1) smoking can severely impair insulin action by increasing insulin response and C-peptide activity to a glucose load [25]; (2) the ingredient of cigarettes can increase hepatic lipase activity, which is associated with elevated insulin resistance [26]; (3) cigarette smoke can damage the pancreas by impairing *β*-cell function and insulin receptor sensitivity [27]; (4) tobacco smoke can activate systemic inflammation which increases the risk of type 2 diabetes [28]; and (5) smoking drastically elevates biomarkers of oxidative stress [29], which plays a role in the development of diabetes mellitus [5]. As aforementioned, GGT is also a biomarker of oxidative stress. In our study, median GGT levels in nonsmokers were obviously lower than those in ex- or current-smokers (13 vs 19 in male and 13 vs 18 in female). Maybe this can explain why GGT is not associated with the risk of diabetes mellitus in ex- or current-smokers after adjusting smoking status. The similar result was also observed in different drinking subgroups. Although numerous studies have reported that the GGT level is used as a biomarker of higher alcohol intake [30, 31], the reason why GGT is not associated with the risk of diabetes mellitus in drinkers is unclear and needs further study. In a 12-year follow-up study, it showed that HRs were higher in participants who drank more than 20 g/day (1.9 (1.1, 3.3)) than in those who drank less than 20 g/day (1.3 (0.9, 1.8)) [8]. This result was opposite to ours, and more studies need to be performed.

The strengths of our study included the relatively large population-based cohort study, and adjusting the influence of fatty liver as serum GGT was closely related to fatty infiltration of the liver and considered as a surrogate marker of NAFLD [32]. Besides, we excluded participants with known liver disease such as viral hepatitis, which might affect our results. Our study also has some limitations. First, we lacked the data on hepatic insulin resistance, which was positively correlated with GGT [33] and would attenuate the hazard rations. However, other studies that adjusted insulin resistance still reported significant associations between elevated GGT levels and incident diabetes mellitus [19, 34]. Second, OGTT was not performed in this study, and the incidence of diabetes mellitus might have been underestimated. In addition, all blood tests including GGT were based on a single measurement, which might underestimate the strength of the associations if regression dilution bias existed.

## 5. Conclusions

In conclusion, an increase of serum GGT levels was independently associated with a higher incidence of diabetes mellitus in this prospective study during 5.4-year follow-up of Japanese participants. A positive curvilinear association between GGT and incident diabetes mellitus was present, with a saturation effect predicted at 24 IU/L of serum GGT levels.

## Figures and Tables

**Table 1:** Baseline characteristics of participants in the NAGALA study by categories of serum GGT levels, 2004–2015.

Variable	All participants	Serum GGT quintiles					*P* value
		*Q*1 (≤10 IU/L)	*Q*2 (11–12 IU/L)	*Q*3 (13–16 IU/L)	*Q*4 (17–24 IU/L)	*Q*5 (≥25 IU/L)	
Participants (*n*)	15464	2885	2328	3674	3304	3273	
Age (years)	43.71 ± 8.90	42.18 ± 8.49	42.60 ± 8.65	43.56 ± 9.18	44.36 ± 9.00	45.35 ± 8.66	<0.001
Female	7034 (45.49%)	2467 (85.51%)	1642 (70.53%)	1751 (47.66%)	743 (22.49%)	431 (13.17%)	<0.001
Smoking status							<0.001
Never	9031 (58.40%)	2340 (81.11%)	1707 (73.32%)	2246 (61.13%)	1475 (44.64%)	1263 (38.59%)	
Past	2952 (19.09%)	277 (9.60%)	278 (11.94%)	632 (17.20%)	840 (25.42%)	925 (28.26%)	
Current	3481 (22.51%)	268 (9.29%)	343 (14.73%)	796 (21.67%)	989 (29.93%)	1085 (33.15%)	
Alcohol intake	1 (0–60.5)	1 (0–4.2)	1 (0–12)	1 (0–40)	12 (1–90)	60 (1–154)	<0.001
Habit of exercise							0.001
Never	12755 (82.48%)	2399 (83.15%)	1946 (83.59%)	2975 (80.97%)	2684 (81.23%)	2751 (84.05%)	
≥1/week	2709 (17.52%)	486 (16.85%)	382 (16.41%)	699 (19.03%)	620 (18.77%)	522 (15.95%)	
BMI (kg/m^2^)	22.12 ± 3.13	20.67 ± 2.40	21.07 ± 2.66	21.66 ± 2.88	22.83 ± 3.12	23.93 ± 3.21	<0.001
WC (cm)	76.47 ± 9.11	70.54 ± 7.27	72.79 ± 7.53	75.37 ± 8.08	79.37 ± 8.35	82.63 ± 8.53	<0.001
SBP (mmHg)	114.50 ± 14.97	107.11 ± 12.95	109.68 ± 13.44	113.76 ± 14.31	117.66 ± 14.08	122.08 ± 14.84	<0.001
DBP (mmHg)	71.58 ± 10.50	66.29 ± 8.90	67.97 ± 9.32	70.96 ± 9.93	73.90 ± 9.98	77.18 ± 10.36	<0.001
Fatty liver	86 (2.98%)	130 (5.58%)	436 (11.87%)	828 (25.06%)	1261 (38.53%)	86 (2.98%)	<0.001
ALT (IU/L)	17 (13–23)	12 (10–15)	14 (11–17)	16 (13–20)	19 (15–25)	26 (20–37)	<0.001
AST (IU/L)	17 (14–21)	15 (12–17)	16 (13–19)	17 (14–20)	18 (15–22)	21 (18–26)	<0.001
GGT (IU/L)	15 (11–22)	9 (8–10)	12 (11–12)	14 (13–15)	20 (18–22)	35 (28–48)	<0.001
TC (mmol/L)	5.13 ± 0.86	4.90 ± 0.83	4.96 ± 0.82	5.06 ± 0.83	5.21 ± 0.87	5.43 ± 0.86	<0.001
HDL (mmol/L)	1.46 ± 0.40	1.59 ± 0.37	1.56 ± 0.39	1.49 ± 0.41	1.37 ± 0.40	1.33 ± 0.39	<0.001
TG (mmol/L)	0.73 (0.50–1.12)	0.54 (0.40–0.75)	0.59 (0.41–0.82)	0.69 (0.47–0.99)	0.88 (0.61–1.28)	1.12 (0.76–1.67)	<0.001
FPG (mmol/L)	5.16 ± 0.41	4.95 ± 0.38	5.02 ± 0.39	5.14 ± 0.39	5.27 ± 0.38	5.37 ± 0.38	<0.001
HbA1c (%)	5.17 ± 0.32	5.11 ± 0.32	5.15 ± 0.31	5.18 ± 0.31	5.20 ± 0.32	5.21 ± 0.34	<0.001

Notes: data presented are mean ± SD, median (*Q*1–*Q*3), or *N* (%). Abbreviations: BMI, body mass index; WC, waist circumference; SBP, systolic blood pressure; DBP, diastolic blood pressure; ALT, alanine aminotransferase; AST, aspartate aminotransferase; GGT, gamma-glutamyl transferase; TC, total cholesterol; HDL, high-density lipoprotein cholesterol; TG, triglyceride; FPG, fasting plasma glucose; HbA1c, haemoglobin A1c.

**Table 2:** Association between serum GGT levels and incident diabetes mellitus in the NAGALA study, 2004–2015.

	Nonadjusted	Adjust I	Adjust II	Adjust III
GGT (IU/L)	1.01 (1.01, 1.01)	1.01 (1.01, 1.01)	1.01 (1.00, 1.01)	1.00 (1.00, 1.01)
Serum GGT quintiles				
*Q*1 (≤10 IU/L)	1	1	1	1
*Q*2 (11–12 IU/L)	2.02 (1.13, 3.61)	1.92 (1.07, 3.46)	1.74 (0.97, 3.13)	1.39 (0.77, 2.50)
*Q*3 (13–16 IU/L)	2.49 (1.47, 4.20)	2.22 (1.30, 3.79)	1.81 (1.06, 3.10)	1.15 (0.66, 1.98)
*Q*4 (17–24 IU/L)	5.95 (3.68, 9.60)	5.00 (3.00, 8.34)	3.33 (1.99, 5.58)	1.76 (1.04, 3.00)
*Q*5 (≥25 IU/L)	9.27 (5.83, 14.72)	7.51 (4.54, 12.41)	4.30 (2.56, 7.20)	1.83 (1.06, 3.15)
*P* for trend	<0.0001	<0.0001	<0.0001	0.0097

Notes: data presented are HRs and 95% CIs. Adjust I model adjusts for age and gender; adjust II model adjusts for adjust I + smoking status, alcohol intake, body mass index, and systolic blood pressure; adjust III model adjusts for adjust II + fatty liver, triglycerides, alanine aminotransferase, aspartate aminotransferase, and fasting plasma glucose. Abbreviations: GGT, gamma-glutamyl transferase.

**Table 3:** Threshold effect analysis of GGT on incident diabetes mellitus in the NAGALA study, 2004–2015.

Outcome:	HR (95% CI)	*P* value
One-line linear regression model	1.00 (1.00, 1.01)	0.0922
Two-piecewise linear regression model		
GGT <24	1.04 (1.02, 1.07)	0.0017
GGT ≥24	1.00 (0.99, 1.01)	0.9733
Log-likelihood ratio test	0.003	

Notes: adjusted for age, gender, smoking status, alcohol intake, body mass index, systolic blood pressure, fatty liver, triglycerides, alanine aminotransferase, aspartate aminotransferase, and fasting plasma glucose. Abbreviations: GGT, gamma-glutamyl transferase; HR, hazard ratio; CI, confidence interval.

**Table 4:** Subgroup analyses of the association between serum GGT levels and incident diabetes mellitus in the NAGALA study, 2004–2015.

Confounding factor category	Serum GGT quintiles					*P* for trend	*P* for interaction
	*Q*1 (≤10 IU/L)	*Q*2 (11–12 IU/L)	*Q*3 (13–16 IU/L)	*Q*4 (17–24 IU/L)	*Q*5 (≥25 IU/L)		
Alcohol intake							0.2865
0 g/week	1 (reference)	1.38 (0.50, 3.84)	2.03 (0.86, 4.78)	3.85 (1.67, 8.88)	3.92 (1.60, 9.58)	0.0003	
>0 g/week	1 (reference)	1.30 (0.63, 2.70)	0.89 (0.44, 1.80)	1.29 (0.66, 2.53)	1.28 (0.65, 2.52)	0.3495	

Smoking status							0.0378
Never	1 (reference)	1.89 (0.90, 3.95)	1.40 (0.67, 2.90)	2.60 (1.28, 5.27)	2.57 (1.22, 5.39)	0.0090	
Past	1 (reference)	1.00 (0.22, 4.52)	0.68 (0.17, 2.78)	0.52 (0.13, 2.07)	1.22 (0.33, 4.55)	0.2123	
Current	1 (reference)	0.59 (0.16, 2.24)	0.64 (0.22, 1.82)	1.02 (0.37, 2.81)	0.70 (0.25, 1.98)	0.8382	

BMI (kg/m^2^)							0.1647
<25	1 (reference)	1.03 (0.52, 2.03)	1.11 (0.60, 2.06)	1.87 (1.04, 3.37)	1.78 (0.96, 3.29)	0.0115	
≥25	1 (reference)	5.64 (1.23, 25.84)	3.37 (0.77, 14.78)	4.58 (1.07, 19.64)	5.46 (1.26, 23.61)	0.0356	

WC (cm)							0.4253
<90 in men, <80 in women	1 (reference)	1.11 (0.58, 2.14)	0.93 (0.50, 1.71)	1.52 (0.85, 2.73)	1.59 (0.87, 2.90)	0.0325	
≥90 in men, ≥80 in women	1 (reference)	6.47 (0.81, 51.77)	7.03 (0.93, 53.42)	9.84 (1.32, 73.59)	9.87 (1.31, 74.44)	0.0189	

Notes: adjusted for age, gender, smoking status, alcohol intake, body mass index, systolic blood pressure, fatty liver, triglycerides, alanine aminotransferase, aspartate aminotransferase, and fasting plasma glucose. Abbreviations: GGT, gamma-glutamyl transferase; BMI, body mass index; WC, waist circumference.

## Data Availability

Data can be downloaded from the DRYAD database (http://www.Datadryad.org).
